# Synthesis of Ti_4_O_7_/Ti_3_O_5_ Dual-Phase Nanofibers with Coherent Interface for Oxygen Reduction Reaction Electrocatalysts

**DOI:** 10.3390/ma13143142

**Published:** 2020-07-14

**Authors:** Ruyue Shi, Ying Huang, Miaoran Li, Ying Zhu, Xuexia He, Ruibin Jiang, Zhibin Lei, Zonghuai Liu, Jie Sun

**Affiliations:** 1 Shaanxi Key Laboratory for Advanced Energy Devices, Shaanxi Engineering Laboratory for Advanced Energy Technology, School of Materials Science and Engineering, Shaanxi Normal University, 620 West Chang’an Street, Xi’an 710119, China; sry@snnu.edu.cn (R.S.); huangying@snnu.edu.cn (Y.H.); lmran9502@snnu.edu.cn (M.L.); yingzhu@snnu.edu.cn (Y.Z.); xxhe@snnu.edu.cn (X.H.); rbjiang@snnu.edu.cn (R.J.); zblei@snnu.edu.cn (Z.L.); zhliu@snnu.edu.cn (Z.L.); 2 Key Laboratory of Applied Surface and Colloid Chemistry (MOE), Shaanxi Normal University, 620 West Chang’an Street, Xi’an 710119, China

**Keywords:** Ti_n_O_2n−1_ phases, interface, oxygen reduction reaction

## Abstract

Electrocatalysts play an important role in oxygen reduction reaction (ORR) in promoting the reaction process. Although commercial Pt/C exhibits excellent performance in ORR, the low duration, high cost, and poor methanol tolerance seriously restrict its sustainable development and application. Ti_n_O_2n−1_ (3 ≤ *n* ≤ 10) is a series of titanium sub-oxide materials with excellent electrical conductivity, electrochemical activity, and stability, which have been widely applied in the field of energy storage and catalysis. Herein, we design and synthesize Ti_4_O_7_/Ti_3_O_5_ (T4/T3) dual-phase nanofibers with excellent ORR catalytic performance through hydrothermal growth, which is followed by a precisely controlled calcination process. The H_2_Ti_3_O_7_ precursor with uniform size can be first obtained by optimizing the hydrothermal growth parameters. By precisely controlling the amount of reducing agent, calcination temperature, and holding time, the T4/T3 dual-phase nanofibers with uniform morphology and coherent interfaces can be obtained. The orientation relationships between T4 and T3 are confirmed to be ${\left[001\right]}_{T3}//{\left[031\right]}_{T4}$, ${\left(100\right)}_{T3}//{\left(92\bar{6}\right)}_{T4}$, and ${\left(010\right)}_{T3}//{\left(1\bar{2}6\right)}_{T4}$, respectively, based on comprehensive transmission electron microscopy (TEM) investigations. Furthermore, such dual-phase nanofibers exhibit the onset potential and half-wave potential of 0.90 V and 0.75 V as the ORR electrocatalysts in alkaline media, respectively, which illustrates the excellent ORR catalytic performance. The rotating ring-disk electrode (RRDE) experiment confirmed the electron transfer number of 3.0 for such catalysts, which indicates a mixture of two electron and four electron transfer reaction pathways. Moreover, the methanol tolerance and cycling stability of the catalysts are also investigated accordingly.

## 1. Introduction

The development of new clean energy technologies is imperative in recent years due to the serious energy crisis and environmental pollution [1,2,3]. As the two most promising new energy technologies, fuel cells and metal-air batteries possess excellent safety and sustainability, which has attracted more attention in recent years [4,5]. These two devices enjoy the same cathodic oxygen reduction reaction: $O_{2}+4H^{+}+4e^{-}\to 2H_{2}O$, which is the key reaction to improve the performance and energy conversion efficiency [6,7,8]. However, the complex reaction path of oxygen reduction leads to the formation of more intermediates, high reaction activation energy, slow kinetic rate, and more. In this case, the efficiency of the oxygen reduction reaction becomes a big challenge affecting the performance of the energy conversion devices [9,10]. Therefore, plenty of research studies have been carried out on the design and synthesis of various Oxygen Reduction Reaction (ORR) electrocatalysts in recent years [11,12,13,14].

According to previous research works, electrocatalysts with excellent performance should contain an appropriate electron number in *d* orbital to possess relevant suitable adsorption energy [4]. Currently, Pt exhibits the best oxygen reduction performance among all transition metals because its binding energy with oxygen atoms is neither too strong nor too weak, which means it can both break the O–O bonds and allow the oxygen species to be adsorbed on the surface and become reduced to water in the subsequent reaction at the same time [15,16,17,18]. On the other hand, the support for the catalysts is another important part, which is crucial for the final performance. Normally, carbon materials are widely applied as Pt supports due to their excellent electrical conductivity and large specific surface area. However, the corrosion of such carbon supports is normally unavoidable during cycling, especially under high current potential, which leads to the dissolution and recrystallization of Pt particles, which further deteriorate the catalytic performance [19,20]. Based on the above-mentioned shortcomings of the Pt/C catalyst, the development of novel non-Pt-based ORR electrocatalysts is one of the solutions to solve the problems.

Among all the non-platinum candidate materials available for ORR catalysts, Magnéli phase titanium sub-oxides (Ti_n_O_2n−1_, 3 ≤ *n* ≤ 10) have received extensive attention due to their high electrical conductivity and chemical stability. Such materials possess a special crystal structure with plenty of periodic crystallographic shear (CS) planes associated with the *n* value. Ti_4_O_7_ and Ti_3_O_5_ are the two most widely used phases not only as the supports of Pt catalyst, but also directly utilized in ORR [3,21]. This is because the mixed valence state of metal ions in metal oxides has been proved to significantly promote the ORR kinetics [22]. On the other hand, the introduction of coherent interfaces between different Ti_n_O_2n−1_ phases may further improve the catalytic effect and cycling stability because the stronger combination between the two phases will lead to the shortened diffusion path of the intermediate during ORR [23].

Based on the above concepts, we designed and synthesized Ti_4_O_7_/λ-Ti_3_O_5_ (T4/T3) dual-phase nanofibers through a hydrothermal reaction, which was followed by a precisely controlled calcination process for the ORR electrocatalyst. By controlling the filling amount of the reactant and hydrothermal temperature, the H_2_Ti_3_O_7_ precursor with uniform growth morphology can be obtained. The polydopamine (PDA) was coated on the surface of H_2_Ti_3_O_7_ nanofibers as the reducing agent for further carbothermal reduction reaction (CRR) process. The phase composition and morphology of the product can be controlled successfully by adjusting the amount of reducing agent, calcination temperature, and holding time. Moreover, the interface structure in the obtained T4/T3 nanofibers was also investigated through comprehensive TEM analysis. The ORR performance of such dual-phase catalysts were evaluated through cyclic voltammetry (CV) and linear sweep voltammetry (LSV) methods. All the catalytic performances are compared with that of commercial Pt/C at the same time.

## 2. Materials and Methods

### 2.1. Synthesis of H_2_Ti_3_O_7_ Precursor Nanofibers

The H_2_Ti_3_O_7_ nanofibers were synthesized by using the previous reported methods [24]. Specifically, 120 g NaOH and 10.7 g of Ti(SO_4_)_2_ were separately dissolved into 200 mL and 80 mL distilled water, which was followed by magnetic stirring until the formation of homogenous solution. Then the two previously mentioned solutions were mixed together. This was followed by 3 h stirring and transferred to 200-mL Teflon-lined stainless-steel reactor. The filling amount of the reactant as well as the reaction temperature were modified from 50% to 80% and 160 °C to 200 °C to illustrate these effects on the growth morphology of the precursor. After a 48-h reaction and being naturally cooled to room temperature, the white precipitate was collected by centrifugation and filtration. Such a precipitate was then washed by distilled water and ethanol successively. After being dried at 60 °C in the oven, the Na_2_Ti_3_O_7_ nanofibers can be obtained. The obtained Na_2_Ti_3_O_7_ nanofibers were then dispersed to 1 mol L^−1^ hydrochloric acid and stirred for 2 h to promote the ion exchange reaction occur sufficiently. After repeated washing with distilled water and ethanol to pH = 7, the products were separated through centrifugation and dried at 60 °C in an oven to obtain the H_2_Ti_3_O_7_ precursor nanofibers.

### 2.2. Synthesis of Ti_4_O_7_/Ti_3_O_5_ Dual Phase Nanofibers

The obtained H_2_Ti_3_O_7_ precursor nanofibers were first dispersed to a buffer solution composed of trizma base and hydrochloric acid with pH = 8~9. Then a certain proportion of PDA was added (PDA:H_2_Ti_3_O_7_ (mass ratio) = 0.5:1, 1:1, 1.5:1 and 2:1). This was followed by stirring at room temperature for 24 h. The obtained brown precipitate was collected by centrifugation and washed several times with distilled water and ethanol. After being dried at 60 °C in the oven, the PDA-coated H_2_Ti_3_O_7_ nanofibers can be obtained. Such nanofibers were then put into the tube furnace and calcinated at 950–1200 °C with different holding times from 10 min to 60 min in order to obtain nanofibers with different Ti_n_O_2n−1_ phase compositions.

### 2.3. Structural Characterization of the Nanofibers

The X-ray diffractometer (Rigaku D/Max-3c, Rigaku, Tokyo, Japan) with Cu K_α_ radiation (*λ* = 0.154 nm) is employed to analyze the phase composition of the nanofibers. The working voltage and current were set to be 40 kV and 15 mA, respectively. The scan range was set from 5° to 80° with the speed of 5° min^−1^. A field-emission scanning electron microscope (FESEM, SU8020, Hitachi, Tokyo, Japan) and transmission electron microscope (JEM-2800, JEOL, Tokyo, Japan) were utilized to characterize the morphologies and structures of the nanofibers. The EDX mapping experiment was also conducted on the same TEM. All the TEM images were processed on Gatan^®^ DigitalMicrograph software (Gatan, Inc. Pleasanton, CA, USA).

### 2.4. Electrochemical Performance Characterization

Electrochemical performance of the catalysts was evaluated on a three-electrode system in O_2_-saturated alkaline solution (0.1 M KOH) under room temperate with a rotating disc as the working electrode, platinum sheet, and saturated electrode Ag/AgCl as the counter and reference electrode, respectively. The T4/T3 catalysts (10 mg), distilled water (735 μL), ethyl alcohol (185 μL), and Nafion solution (80 μL) were mixed together to form a homogenous ink through ultrasonic dispersion. Then, 10-μL ink was deposited on a glassy carbon electrode with a 5-mm diameter and dried in air. Lastly, the catalytic activity of the catalysts was evaluated by cyclic voltammetry (CV) with the scan speed of 50 mV/s as well as linear sweep voltammetry (LSV) at different rotation speeds (225, 400, 625, 900, 1225, 1600, 2025, and 2500 rpm) with the scan speed of 10 mV/s by using the rotating disk electrode (RDE), respectively. The electron transfer number (*n*) of the catalysts was also determined through the rotating ring disk electrode (RRDE) testing method in which the peroxide species produced at the disk electrode were detected by the ring electrode. The electron transfer number could be calculated from the ratio of the ring current (*I_r_*) and the disk current (*I_d_*) by using the following equation.

$$n=\frac{4I_{d}}{I_{d}+\frac{I_{r}}{N}}$$

where *N* is the collection efficiency (0.37) of the ring electrode.

## 3. Results and Discussions

### 3.1. Morphology and Phase Evolution of Ti_n_O_2n−1_ Dual-Phase Nanofibers

During the hydrothermal reaction, the filling amount of the reactant has been confirmed to exhibit an important effect on the growth morphology of the final products [25]. Basically, a higher filling amount leads to a larger pressure inside the reactor, which leads to the change of reactants solubility and the pH value, and, thereby, affects the reaction rate and the morphology of the products [26]. As shown in Figure S1, it is noticed that the size of the nanofibers becomes more uniform with an increased diameter when the filling amount of the reactor changed from 50% to 80%. In particular, the H_2_Ti_3_O_7_ nanofibers with the average diameter of around 130 nm can be obtained when the filling amount of the reactant reaches 70%. The precursor synthesized under this filling amount is then used in the subsequent calcination process. In addition, Figure S2 illustrates the morphologies of the obtained H_2_Ti_3_O_7_ nanofibers prepared under different hydrothermal reaction temperatures (160 °C, 180 °C, and 200 °C). As seen from the figure, no significant fibrous morphology of the product can be observed when the hydrothermal temperature is 160 °C. With the increase of the reaction temperature, the fiber morphology starts to appear with the increased length to diameter ratio. Specifically, uniform H_2_Ti_3_O_7_ nanofibers can be obtained when the reaction temperature reaches 200 °C.

**Figure 1:**
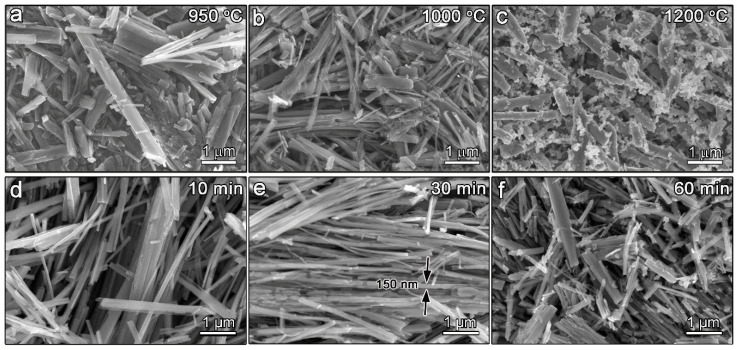
SEM images of T4/T3 nanofibers with (**a**–**c**) different calcination temperatures and (**d**–**f**) holding times.

By carrying out the Carbothermal Reduction Reactiop (CRR) on the H_2_Ti_3_O_7_ precursor nanofibers, Ti_n_O_2n−1_ phases can be obtained accordingly. Figure 1 are the SEM images illustrating the effect of calcination temperature and holding time on the growth morphology of the final products. According to our previous results, the formation temperature of T4 and T3 phases during CRR is around 900–1200 °C [27,28]. Therefore, the calcination temperatures are set to be 900 °C, 1000 °C, and 1200 °C, respectively. Seeing from Figure 1a–c, it is noticed that the length to diameter ratio of the nanofiber decrease with the increment of the calcination temperature. Meanwhile, the morphology gradually changes from the lath shape to the round bar. Specifically, serious grain coarsening can be observed from Figure 1c, which is definitely harmful to its catalytic performance. The effect of the holding time on the final morphology of the products are also investigated through SEM. There is no significant morphology variation when the holding time changed from 10 min to 30 min, as shown in Figure 1d,e. The diameter of the nanofibers reaches around 150 nm when preserved at 1000 °C for 30 min. However, a clear diameter increase of the nanofibers can be observed when the holding time reached 60 min, as indicated in Figure 1f. In addition, the morphology changes from lath shape to the round rod occur with the increment of holding time.

Since the grain coarsening is non-ignorable during the CRR, the introduced PDA not only acts as the reducing agent, but also restrains the grain growth through physical confinement during the calcination process. Therefore, the effect of PDA dosage on the morphology and phase composition of the products is also investigated accordingly, as shown in Figures S3 and S4. In this case, the calcination temperature is selected to be 1000 °C with the holding time of 30 min. As seen from the figure, the grain coarsening of products cannot be restrained effectively after high temperature calcination when the weight ratio of PDA and H_2_Ti_3_O_7_ is below 1:1. In addition, the phase composition is confirmed to be the mixture of T4, T3, and anatase through XRD analysis, which indicates the insufficient amount of reducing agent. When increasing the PDA:H_2_Ti_3_O_7_ mass ratio to 1.5:1, the morphology of the product has not changed much when compared with that before the calcination process. Meanwhile, the existence of both T4 and T3 phases can be confirmed through the XRD spectra in Figure S4. Furthermore, it is clear to observe the residual carbon layer at the surface of the fiber when the PDA:H_2_Ti_3_O_7_ mass ratio reaches 2:1, which indicates the reductant is excessive under such a condition. Therefore, the PDA:H_2_Ti_3_O_7_ mass ratio is selected to be 1.5:1 to perform the CRR in this work.

**Figure 2:**
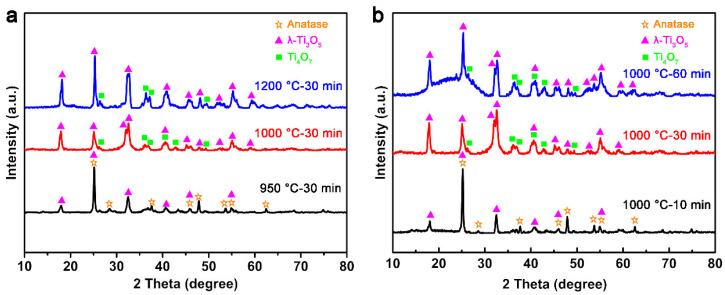
XRD images of nanofibers with (**a**) a different calcination temperature and (**b**) holding time.

The phase evolutions of the products during the carbothermal reduction process under different calcination temperatures and holding times are also investigated by XRD, as shown in Figure 2. When the calcination temperature reaches 950 °C, the XRD spectrum of the product reveals the coexistence of the anatase (JSCPD# 84-1285) and λ-Ti_3_O_5_ (T3) (JCSPD# 82-1137) phase. This is consistent with the previous reported results indicating T3 existed as the transition phase during the transformation from TiO_2_(B) to anatase [29]. When the temperature reaches 1000 °C, Ti_4_O_7_ (T4) (JCSPD# 71-0574) can be found from the XRD spectrum, which indicates the continual process of the CRR at higher temperature. In addition, the peaks representing the anatase phase disappear at this stage, and leaves only T4 and T3 phases in the product. Some of the characteristic peaks of T4 disappear when the calcination temperature reaches 1200 °C, which illustrates the transformation from T4 to T3 occurs at this stage. Combined with the SEM results, 1000 °C can be selected as the calcination temperature to obtain the T4/T3 dual-phase nanofibers.

The effect of holding time on the phase composition of the nanofibers is also investigated through XRD analysis. As seen from Figure 2b, the phase composition of the product is anatase and T3 when calcinated at 1000 °C for 10 min. As the holding time continues to be extended, T4 starts to appear in the product. Such phase compositions remain until the holding time reaches 60 min. Considering the morphology of the product at the same time, it can be concluded that the optimized synthetic process for T4/T3 dual-phase nanofibers through carbothermal reduction reaction is calcinated at 1000 °C for 30 min.

### 3.2. TEM Analysis of T4/T3 Interface Structure

**Figure 3:**
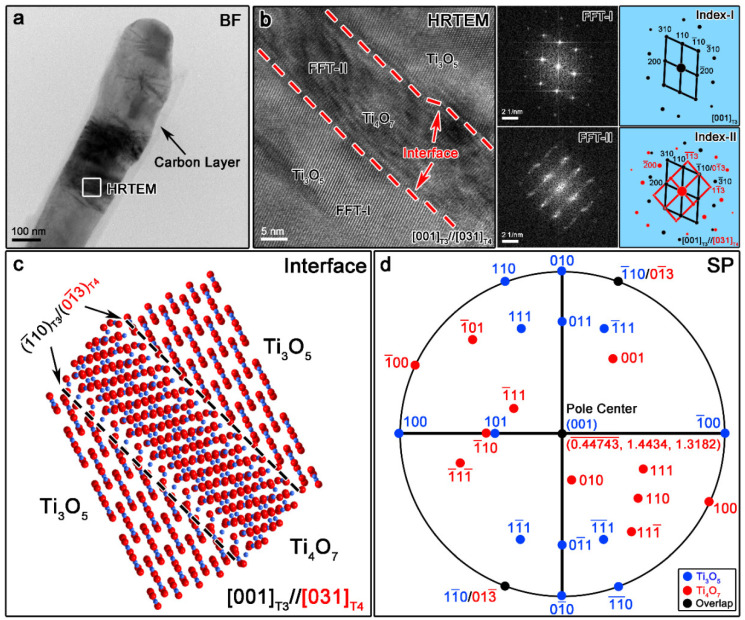
The TEM Bright Field (BF) (**a**) and HRTEM image (**b**) of one T4/T3 dual-phase nanofiber suggest the existence of the carbon layer and the semi-coherent nature of the interface. The interface structure model (**c**) and stereographic projection diagram of planes (**d**) also illustrate the detailed interface crystallographic features.

Figure 3 illustrates the TEM characterization results of the T4/T3 dual phase nanofibers. As seen from the TEM Bright Field (BF) image in Figure 3a, a different contrast is clearly observed on the fiber, which indicates the existence of multi-phases. The fibrous morphology is also maintained well after the calcination, which suggests the excellent physical confinement effect of the coated PDA layer. Meanwhile, an amorphous carbon shell with the thickness of around 10 nm is also found coated at the surface of the nanofiber, which indicates the excessive reducing agent introduced previously. Taken the HRTEM image from the marked region in Figure 3a, significant different lattice fringes can be seen clearly on both sides of the red dashed lines. This effect is considered to be different phases, as indicated in Figure 3b. Through the Fast Fourier Transform (FFT) image taken from the corresponding regions, two sets of diffraction patterns can be indexed as T3 and T4 phases with the zone axis of ${\left[001\right]}_{T3}$ and ${\left[031\right]}_{T4},$ respectively. Meanwhile, the interface between the two phases is found parallel to ${\left(\bar{1}10\right)}_{T3}/{\left(0\bar{1}3\right)}_{T4}$ due to the similar d-spacing of the two lattice planes ($d_{\left(\bar{1}10\right)T3}=0.3535\;\mathrm{nm}$, $d_{\left(0\bar{1}3\right)T4}=0.3710\;\mathrm{nm}$). Furthermore, there are plenty of defects that can be seen from the T4 phase, which may be due to the internal stress caused by dramatic changes in the crystal structure from T4 to T3. The interface model can be drawn based on the HRTEM results, as shown in Figure 3c. As seen from the figure, the interface exhibits semi-coherent features, which may be due to the relatively large crystal structure difference between T3 and T4. With the help of Stereographic Projection software, the orientation relationship (OR) between the two phases can be summarized by the formulas below [30].

$${\left[001\right]}_{T3}//{\left[031\right]}_{T4}$$

$${\left(100\right)}_{T3}//{\left(92\bar{6}\right)}_{T4}$$

$${\left(010\right)}_{T3}//{\left(1\bar{2}6\right)}_{T4}$$

Such OR can also be drawn intuitively in the stereographic projection of planes, as shown in Figure 3d. In this case, the pole center is set to be ${\left(001\right)}_{T3}/{\left(\bar{0.44743},\;1.4434,\;1.3182\right)}_{T4}$, which corresponds to the normal plane of ${\left[001\right]}_{T3}//{\left[031\right]}_{T4}$. In addition, it is found that only limited parallel relations can be observed from the figure, which further proved the semi-coherent feature of the interface. The existence of such an interface is helpful to the enhancement of ORR catalytic performance as well as the cycling stability as it may not only shorten the diffusion distance of the reaction intermediates, but also provide strong catalytic activity sites.

### 3.3. Electrochemical Performance of T4/T3 Dual-Phase Catalysts

The loading amount of the catalysts has a very important effect on its ORR catalytic activity. Therefore, the Linear Sweep Voltammetry (LSV) curves of the catalysts with a different loading amount are first collected, as shown in Figure S5. As seen from the figure, it is found that the onset potential, half-wave potential, and limiting current density are all optimized with the increase of catalysts’ loading from 2 mg to 10 mg. However, the two potentials that appear slightly decrease when the loading amount reaches 12 mg. Under this condition, a 10-mg catalyst is selected to prepare the ink for the following ORR performance test, which is equivalent to 0.5 mg/cm^2^.

**Figure 4:**
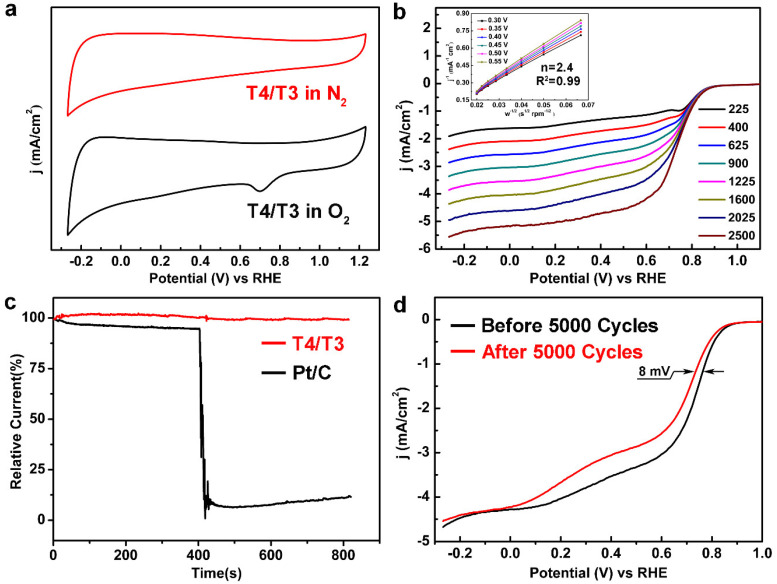
The comparison of the CV curves (**a**) in N_2_ and O_2_-saturated 0.1 M KOH solution confirmed the ORR catalytic activity of T4/T3 dual-phase catalysts. The LSV curves (**b**) at different rotation rates (225, 400, 625, 900, 1225, 1600, 2025, and 2500 rpm) illustrate the initial and half-wave potential of 0.90 V and 0.75 V, respectively. The inset figure shows the Koutecky-Levich plots (j^−1^ vs. w^−1/2^) at different potentials. The i-t chronoamperometric curves (**c**) of T4/T3 and Pt/C catalysts at 0.7 V (vs RHE) in O_2_-saturated 0.1 M KOH solution suggest the excellent methanol resistance of T4/T3 dual-phase catalysts. The turning point represents 6 M methanol added into the solution (at 400 s). Polarization curves of T4/T3 at 0.7 V (vs RHE) in O_2_-saturated 0.1 M KOH solution at a rotating rate of 1600 rpm before and after 5000 cycles (**d**) suggest excellent stability of such catalysts.

The cyclic voltammetry (CV) and rotating disk electrode (RDE) tests are employed to evaluate the ORR activity of T4/T3 dual-phase catalysts, as shown in Figure 4. Seeing from Figure 4a, it is noticed that no apparent oxygen reduction peaks can be observed in N_2_-saturated KOH solution for the T4/T3 dual-phase electrocatalysts, while a clear oxygen reduction peak appears under the O_2_-saturated KOH solution at the scan rate of 50 mV/s. The reduction potential is confirmed to be 0.70 V, which is very close to the value of 0.80 V for commercial Pt/C catalysts (Johnson Matthey Company, London, UK). The onset and half-wave potential of T4/T3 catalysts are confirmed to be 0.90 V and 0.75 V through the linear sweep voltammetry (LSV) curve at the scan rate of 10 mV/s under different rotation speeds, as shown in Figure 4b. Such ORR catalytic activity is very close to that of Pt/C catalysts, whose onset potential and half-wave potential are determined to be 0.99 V and 0.82 V, as shown in Figure S6. Furthermore, the electron transfer number can be calculated from the slope of the Koutecky-Levich curve. For the situation of T4/T3 dual-phase catalysts, the electron transfer number can be calculated to be 2.4 at the voltage range of 0.30–0.55 V, which indicates the two electron and four electron transfer pathways coexisted, as seen from the inset in Figure 4b [31]. To further illustrate the effect of dual-phases existence on the ORR catalytic activity, the LSV curves of pure T4, T3, and T4/T3 dual-phase nanofibers are collected accordingly, as shown in Figure S7. As seen from the figure, the dual-phase T4/T3 catalyst shows a more positive onset and half-wave potential when compared with that of pure T4 and T3 catalysts, which indicates the existence of dual-phase interfaces has a positive effect on promoting catalytic activity. Such a phenomenon can be attributed to the synergistic catalytic effect between T4 and T3 phases. The presence of the interface is conducive to the diffusion of ORR intermediate products and improves the reaction kinetics at the same time [23]. Table S1 also summarizes the ORR performance of other common catalysts in an alkaline condition, which further proves the excellent performance of the T4/T3 catalysts.

The methanol resistance properties of a commercial Pt/C and T4/T3 dual-phase electrocatalyst are also investigated by adding methanol to the electrolyte during the LSV measurement, as shown in Figure 4c. It is noticed that the relative current of the Pt/C catalyst significantly reduced after adding 6 M methanol into 0.1 M KOH solution at 400 s. In contrast, no clear change on the relative current can be observed for the T4/T3 dual-phase electrocatalyst, which indicates the excellent methanol tolerance during the electrocatalytic process. Taken into account of the cycling stability, although the T4/T3 dual-phase catalysts performs an inferior activity compared to 20% commercial Pt/C catalysts, the half wave potential of T4/T3 dual-phase catalysts changed from 0.75 V to 0.67 V with a negative shift of 8 mV, which is relatively smaller than that of commercial Pt/C catalysts (a negative shift of half wave potential reaches to 18 mV) after 5000 cycles, as shown in Figure S8 and Figure 4d, respectively. This indicates that the T4/T3 dual-phase catalysts exhibit an excellent catalytic stability during long time cycling. The morphology of the catalysts after 5000 cycles has also been characterized by SEM, as shown in Figure S9. Seeing from the figure, no significant morphology change can be observed for the nanofibers, which further illustrates the excellent stability of the catalysts in alkaline solution.

**Figure 5:**
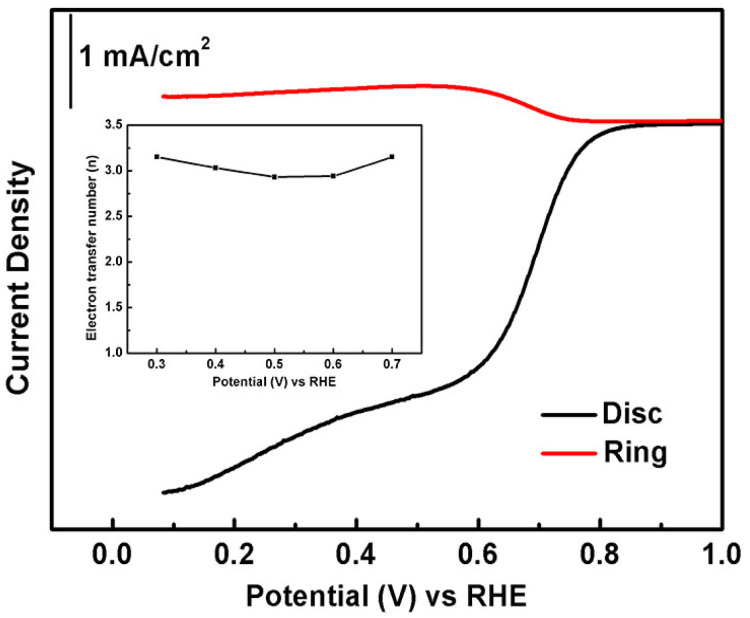
RRDE voltammograms of T4/T3 catalysts in the O_2_-saturated 0.1 M KOH solution with the sweep rate of 5 mV s^−1^ and the rotating rate of 1600 rpm. The ring potential is kept constant at 0.5 V vs. Ag/AgCl. The inset shows the calculated electron transfer number at different potentials.

Lastly, the rotating ring-disk electrode (RRDE) measurements are also carried out to monitor the formation of intermediate products like peroxide species (HO^2−^) during the ORR process. As shown in Figure 5, the current collected at the ring electrode, which corresponds to the amount of HO^2−^, is much smaller than the disk current for T4/T3 nanofiber catalysts. The electron transfer number estimated from the ring and disk currents is about 3.0, which is consistent with the Koutecky–Levich fitting results.

## 4. Conclusions

The T4/T3 dual-phase catalysts were successfully synthesized hydrothermally, which was followed by a precisely-controlled calcination process. The filling amount of the reactor as well as the hydrothermal reaction temperatures were first optimized to be 70% and 200 °C during the synthesis of H_2_Ti_3_O_7_ precursor nanofibers in order to achieve the uniform morphology. In addition, the effects of PDA dosage, calcination temperature, and holding time on the phase composition and growth morphology of the products during CRR are also discussed through XRD and SEM. By setting the PDA:H2Ti3O7 mass ratio as 1.5:1, and calcinated at 1000 °C for 30 min, the T4/T3 dual-phase nanofibers can be obtained. Furthermore, the existence of a semi-coherent interface between the two Ti_n_O_2n−1_ phases is confirmed in one nanofiber with the orientation relationships of ${\left[001\right]}_{T3}//{\left[031\right]}_{T4}$, ${\left(100\right)}_{T3}//{\left(92\bar{6}\right)}_{T4}$, and ${\left(010\right)}_{T3}//{\left(1\bar{2}6\right)}_{T4}$, respectively. Through the LSV and RRDE measurements, the onset and half-wave potential of T4/T3 dual-phase catalysts are confirmed to be 0.90 V and 0.75 V with the electron transfer number of 3.0. Such catalysts are also confirmed to exhibit excellent methanol tolerance and cycling stability at the same time.

## Supplementary Materials

The following are available online at https://www.mdpi.com/1996-1944/13/14/3142/s1. Figure S1: SEM images of the obtained H_2_Ti_3_O_7_ nanofibers under different filling amounts of the reactor. Figure S2: SEM images of the H_2_Ti_3_O_7_ nanofibers under different hydrothermal reaction temperatures. Figure S3: SEM images of the obtained Ti_n_O_2n−1_ nanofibers with different PDA:H_2_Ti_3_O_7_ weight ratio. Figure S4: XRD spectra of the Ti_n_O_2n−1_ nanofibers with different contents of PDA. Figure S5: The effect of different dosage of catalysts on the ORR performance. Figure S6: The ORR catalytic performance of 20% commercial Pt/C in 0.1 M KOH solution. Figure S7: The LSV curves of T4, T3, and T4/T3 catalysts at 1600 rpm. Figure S8: Cycling stability of 20% commercial Pt/C catalysts in 0.1 M KOH solution under 1600 rpm. Figure S9: SEM image of T4/T3 nanofibers after the long time working. Table S1: The comparison of ORR performance of some related non-platinum catalysts.
