# Supplementary material for: Synthesis of Ti4O7/Ti3O5 Dual-Phase Nanofibers with Coherent Interface for Oxygen Reduction Reaction Electrocatalysts
Source: Materials (Basel). 2020 Jul 14;13(14):3142. doi: 10.3390/ma13143142 (PMC7412102; doi:10.3390/ma13143142)
Supplement: Supplementary file 1 [file materials-13-03142-s001.pdf]

materials

MDPI

Synthesis of Ti_4O_7/Ti_3O_5 Dual-Phase Nanofibers with Coherent Interface for Oxygen Reduction Reaction Electrocatalysts

Supplementary Materials

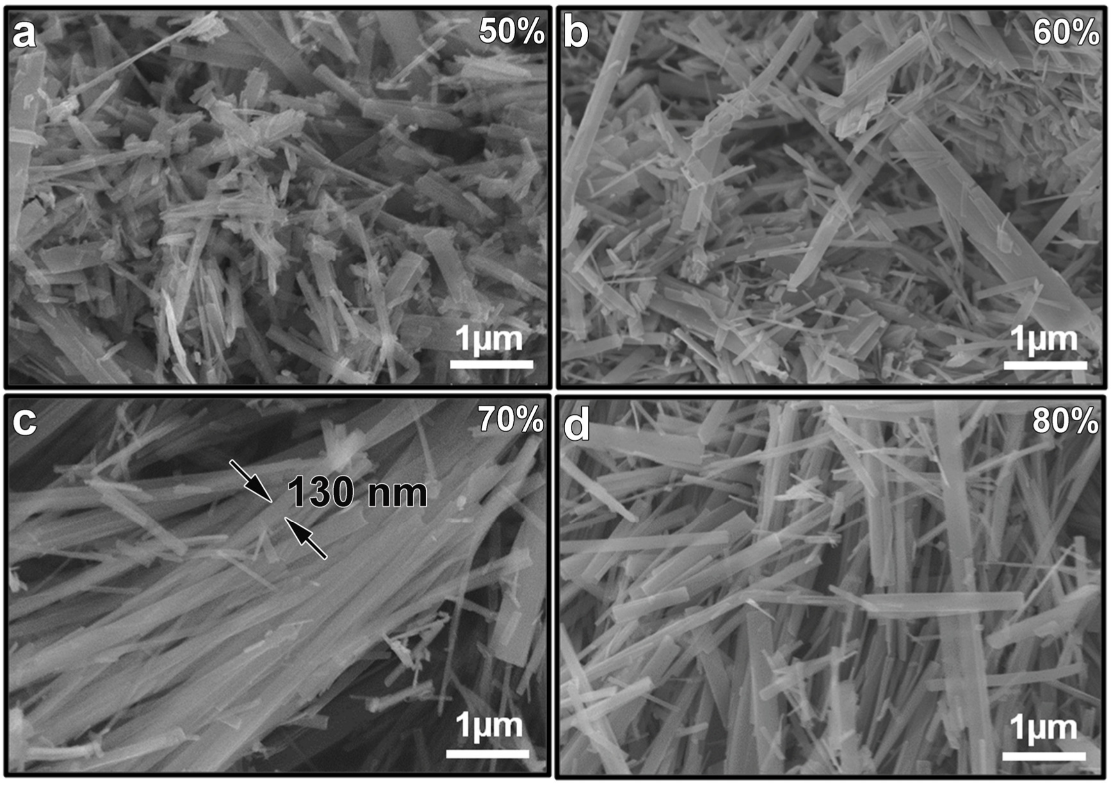

Figure S1. SEM images of the obtained $H_2Ti_3O_7$ nanofibers under different filling amount of the reactor.

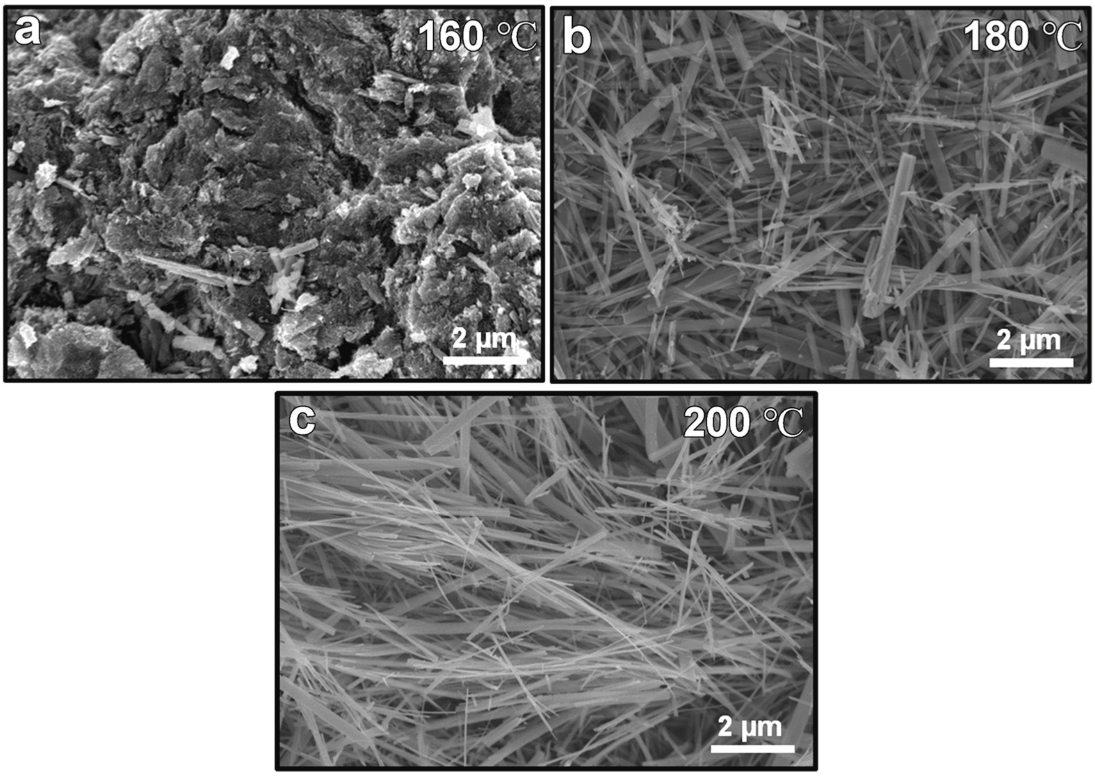

Figure S2. SEM images of the $H_2Ti_3O_7$ nanofibers under different hydrothermal reaction temperatures.

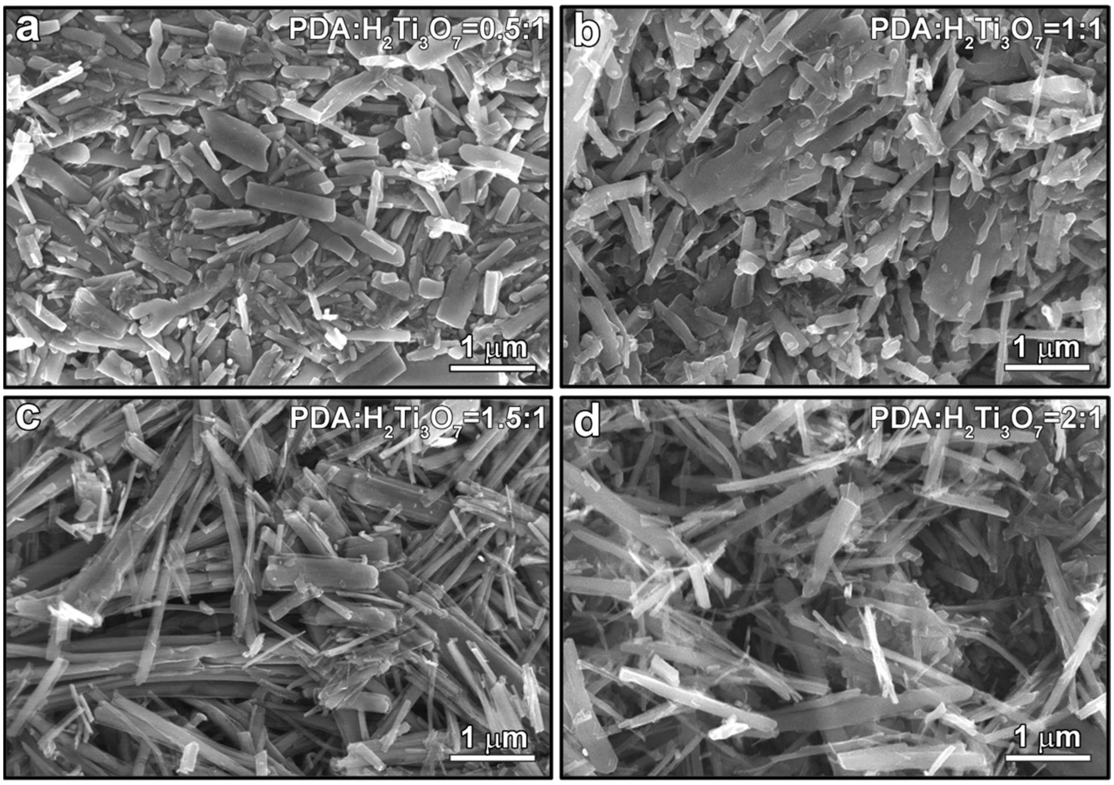

Figure S3. SEM images of the obtained Ti_nO_{2n-1} nanofibers with different PDA: $H_2Ti_3O_7$ weight ratio.

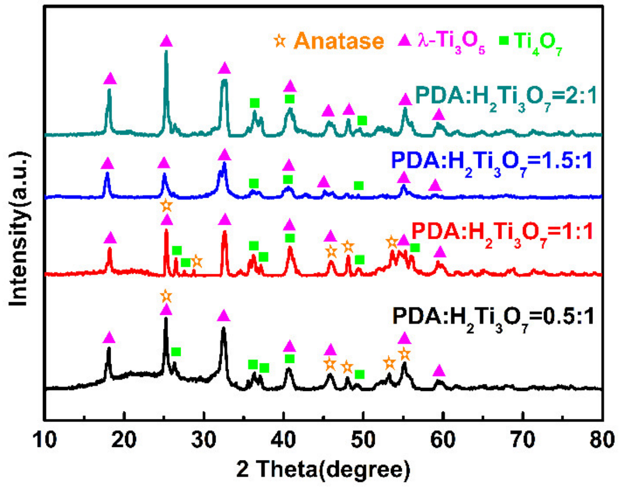

Figure S4. XRD spectra of the Ti_nO_{2n-1} nanofibers with different content of PDA.

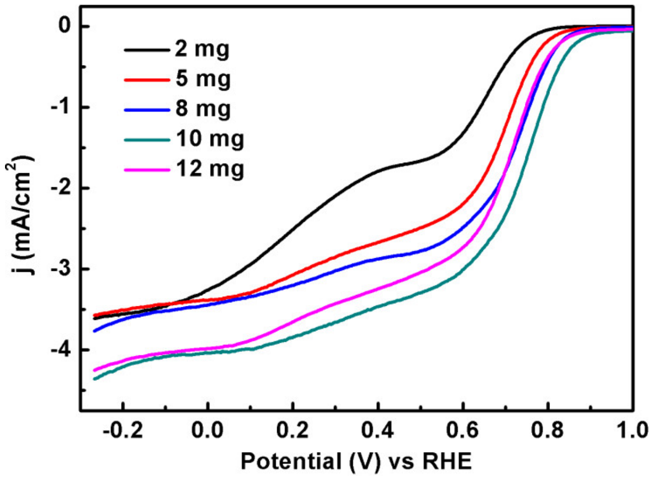

Figure S5. The effect of catalysts loading amount on the ORR performance.

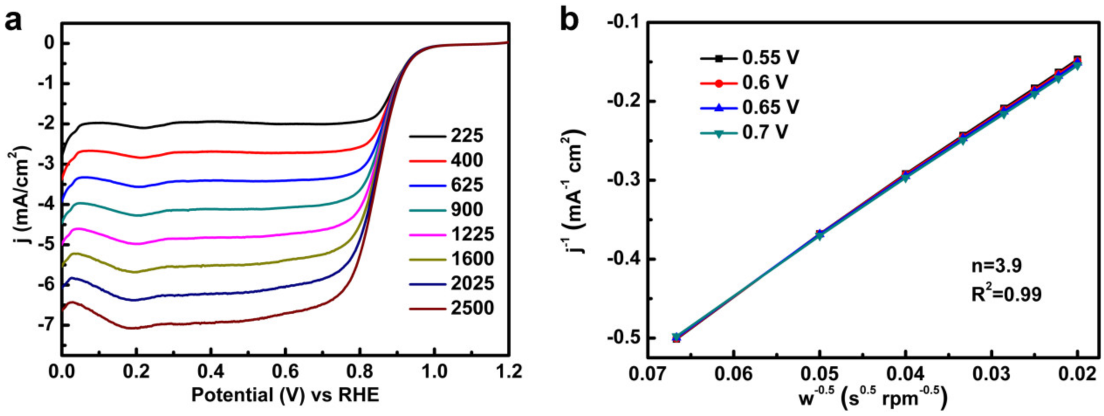

Figure S6. The ORR catalytic performance of 20% commercial Pt/C in 0.1 M KOH solution.

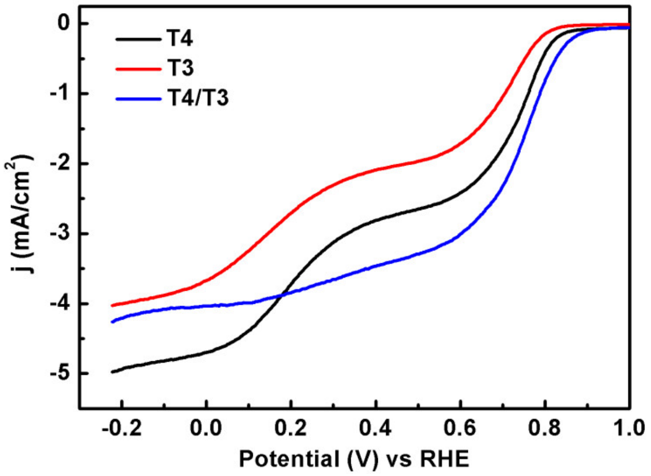

Figure S7. The LSV curves of T4, T3 and T4/T3 catalysts at 1600 rpm.

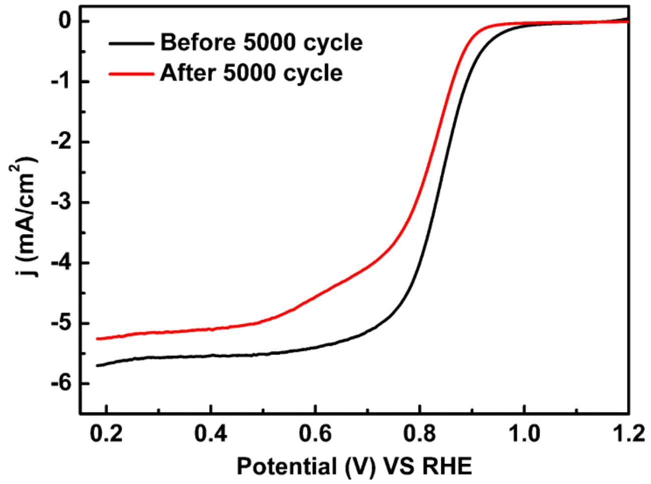

Figure S8. Cycling stability of 20% commercial Pt/C catalysts in 0.1 M KOH solution under 1600 rpm.

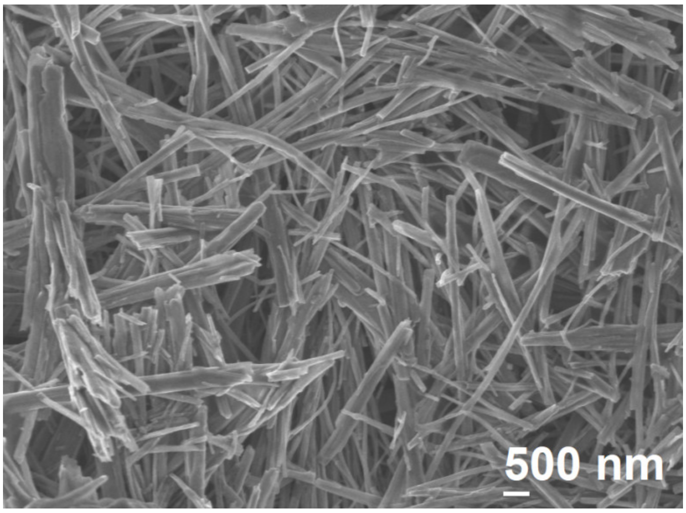

Figure S9. SEM image of T4/T3 nanofibers after the long time working.

Table S1. The comparison of ORR performance of some common non-platinum catalysts in alkaline solution.

Catalysts	Media	Onset Potential (vs. RHE)	Half-wave Potential (vs. RHE)	References
TiO_2/N-doped Carbon	0.1 M KOH	0.76 V	0.65 V	[1]
Co-N-C Nanosheets		0.72 V	0.90 V	[2]
FeNP-C		0.90 V	0.72 V	[3]
Fe-NHHPC900		0.94 V	0.86 V	[4]
T4/T3 catalyst		0.90 V	0.75 V	This work

References

1. Jin, S.; Li, C.; Shrestha, L.K.; Yamauchi, Y.; Ariga, K.; Hill, J.P. Simple fabrication of titanium dioxide/N-doped carbon hybrid material as non-precious metal electrocatalyst for the oxygen reduction reaction. *ACS Appl. Mater. Interfaces* **2017**, *9*, 18782–18789.
2. Lyu, D.; Du, Y.; Huang, S.; Mollamahale, B.Y.; Zhang, X.; Hasan, S.W.; Yu, F.; Wang, S.; Tian, Z.Q.; Shen, P.K. Highly Efficient Multifunctional Co–N–C Electrocatalysts with Synergistic Effects of Co–N Moieties and Co Metallic Nanoparticles Encapsulated in a N-Doped Carbon Matrix for Water-Splitting and Oxygen Redox Reactions. *ACS Appl. Mater. Interfaces* **2019**, *11*, 39809–39819.
3. Hu, Y.; Zhu, J.; Lv, Q.; Liu, C.; Li, Q.; Xing, W. Promotional effect of phosphorus doping on the activity of the Fe-N/C catalyst for the oxygen reduction reaction. *Electrochim. Acta* **2015**, *155*, 335–340.
4. Tian, P.; Wang, Y.; Li, W.; Song, S.; Zhou, S.; Gao, H.; Xu, H.; Tian, X.; Zang, J. A salt induced gelatin crosslinking strategy to prepare Fe-N doped aligned porous carbon for efficient oxygen reduction reaction catalysts and high-performance supercapacitors. *Chin. J. Catal.* **2020**, *382*, 109–120.
